# Predicting Habitual Use of Wearable Health Devices Among Middle-aged Individuals With Metabolic Syndrome Risk Factors in South Korea: Cross-sectional Study

**DOI:** 10.2196/42087

**Published:** 2023-04-06

**Authors:** Jaeyoung Ha, Jungmi Park, Sangyi Lee, Jeong Lee, Jin-Young Choi, Junhyoung Kim, Sung-il Cho, Gyeong-Suk Jeon

**Affiliations:** 1 Department of Public Health Science Graduate School of Public Health Seoul National University Seoul Republic of Korea; 2 Department of Nursing Chodang University Muan Republic of Korea; 3 Institute of Health and Environment Seoul National University Seoul Republic of Korea; 4 Department of Nursing Mokpo National University Muan Republic of Korea

**Keywords:** wearable health devices, HBM, UTAUT2, habitual use, metabolic syndrome, wearables, digital intervention, health promotion, predictors of use, acceptability

## Abstract

**Background:**

Prevention of the risk factors for metabolic syndrome (MetS) in middle-aged individuals is an important public health issue. Technology-mediated interventions, such as wearable health devices, can aid in lifestyle modification, but they require habitual use to sustain healthy behavior. However, the underlying mechanisms and predictors of habitual use of wearable health devices among middle-aged individuals remain unclear.

**Objective:**

We investigated the predictors of habitual use of wearable health devices among middle-aged individuals with risk factors for MetS.

**Methods:**

We proposed a combined theoretical model based on the health belief model, the Unified Technology of Acceptance and Use of Technology 2, and perceived risk. We conducted a web-based survey of 300 middle-aged individuals with MetS between September 3 and 7, 2021. We validated the model using structural equation modeling.

**Results:**

The model explained 86.6% of the variance in the habitual use of wearable health devices. The goodness-of-fit indices revealed that the proposed model has a desirable fit with the data. Performance expectancy was the core variable explaining the habitual use of wearable devices. The direct effect of the performance expectancy on habitual use of wearable devices was greater (β=.537, *P*<.001) than that of intention to continue use (β=.439, *P*<.001), and the total effect estimate of the performance expectancy was 0.909 (*P*<.001), including the indirect effect (β=.372, *P*=.03) on habitual use of wearable devices via intention to continue use. Furthermore, performance expectancy was influenced by health motivation (β=.497, *P*<.001), effort expectancy (β=.558, *P*<.001), and risk perception (β=.137, *P*=.02). Perceived vulnerability (β=.562, *P*<.001) and perceived severity (β=.243, *P*=.008) contributed to health motivation.

**Conclusions:**

The results suggest the importance of the users’ performance expectations for wearable health devices for the intention of continued use for self-health management and habituation. Based on our results, developers and health care practitioners should find better ways to meet the performance expectations of middle-aged individuals with MetS risk factors. They also should generate device use easier and find a way to encourage users’ health motivation, thereby reducing users’ effort expectancy and resulting in a reasonable performance expectancy of the wearable health device, to induce users’ habitual use behaviors.

## Introduction

Metabolic syndrome (MetS) is characterized by the presence of 3 of the following 5 conditions: abdominal obesity, hypertension, hypertriglyceridemia, hyperglycemia, and high serum low-density lipoprotein [1]. The prevalence of MetS is increasing worldwide, leading to a high burden of several diseases, including cardiovascular disease (CVD) [2,3]. In particular, lifestyle changes associated with urbanization and industrialization in the Asia-Pacific region, including South Korea, have led to a remarkable increase in the prevalence of MetS [4-6]. MetS most commonly affects middle-aged individuals. According to the Korean Statistical Information Service, in 2019, overall 57% of individuals with MetS risk factors were aged 40-65 years [7]. Effective management and prevention of MetS in the middle-aged population are essential to reducing the increasing burden of noncommunicable diseases with aging. MetS may lead to adverse health outcomes, such as the development of CVD and even death [8]. However, it is easily modified through behavioral changes in health [9].

Several studies have evaluated the effectiveness of technology-mediated interventions (eg, wearable health devices, smartphone apps, mobile health, and eHealth) to modify the lifestyles of individuals with MetS [10]. Some studies specifically found the positive effects of wearable activity trackers (WATs) on behavioral change compared with conventional intervention through a randomized controlled trial [11] and with other types of technology (eg, mobile apps) [12]. WATs are particularly effective in improving physical activity by monitoring and recording physical activity [13]. A meta-analysis revealed that WAT-based interventions significantly increase physical activity and improve health indicators (waist circumference, systolic blood pressure, and low-density lipoprotein cholesterol level) in individuals with chronic diseases [13]. WATs offer various personalized features that are convenient for users, such as setting individual goals, tracking progress, monitoring health, and providing feedback, which is regarded as essential to motivating lifestyle change [14]. Although specific technical features, such as usability, aesthetics, reliability and accuracy of measured data, and price affect the preference of wearable types, studies suggest that the overall characteristics of wearable devices that offer personalized use have a greater impact on lifestyle changes than specific features [15,16].

For effective self-health management using wearable health devices to manage risk factors for MetS prevention, a more in-depth understanding of the psychosocial process of users from adoption to use of the devices continuously and habitually is required. Studies on the acceptance of the use of wearable devices for health promotion have found that acceptance and abandonment of wearable devices are mediated by social, cognitive, and psychological factors [15]. For example, the technology acceptance model (TAM) suggests that technology acceptance and use are mainly based on perceived usefulness (ie, perceived effectiveness and efficiency) and perceived ease of use (ie, the perceived cost of device use) [17]. Venkatesh et al [18] developed the Unified Theory of Acceptance and Use of Technology (UTAUT) based on 8 models, including TAM and the theory of planned behavior, to identify the drivers of acceptance of technology. The extended version of UTAUT (UTAUT2) includes 3 additional constructs (hedonic motivation, price value, and habit) that make it more user-centered [19].

However, compared to the many theories and models that have developed and advanced on the initial acceptance of wearable health care devices, there is no unified theory or model for the postadoption stage [20]. Some have combined UTAUT2 with other health behavior models to explain the continued use of technology after initial adoption, as it cannot be explained by a single model [21-23]. Wei et al [23] combined the UTAUT2 and the health belief model (HBM) to identify the predictors of adoption and continued use of a diet and fitness mobile app. Many studies of the continued use of mobile platforms have focused on satisfaction, perceived benefit, and trust [20]. Recent studies have evaluated emerging important constructs, such as perceived privacy risk (ie, risk perception) [23], confirmation, and satisfaction [24]. With advancements in the internet and social media, the perceived risk among consumers is an important consideration for fitness mobile apps [25,26]. Some studies have considered differences in individual characteristics, such as self-efficacy [27] or the creative application of the devices to foster environments to make them routine [28]. Furthermore, according to the qualitative studies of habit formation in wearable device usage among older adults [28,29], strong motivation was an important factor for the long-term usage of wearable health care devices for self-health management.

The middle-aged group with risk factors for MetS is likely to have unique factors in adopting and continuing the use of wearable health care devices that differ from those of the general population. This is due to the conflicting predictors of continued device use, such as the finding that long-term use is less likely for individuals with chronic conditions [29] but is more likely for those with strong motivation and a meaningful initiation [28,29]. Therefore, it is necessary to develop a valid theoretical model suitable for devices for individuals aged >40 years with risk factors for MetS. We set up a hypothetical model incorporating strong health motivation, perceived privacy risk, which has risen as an important factor, and habitual use as the behavioral outcome and tested it with empirical data.

## Methods

### Model Construction

**Figure 1:**
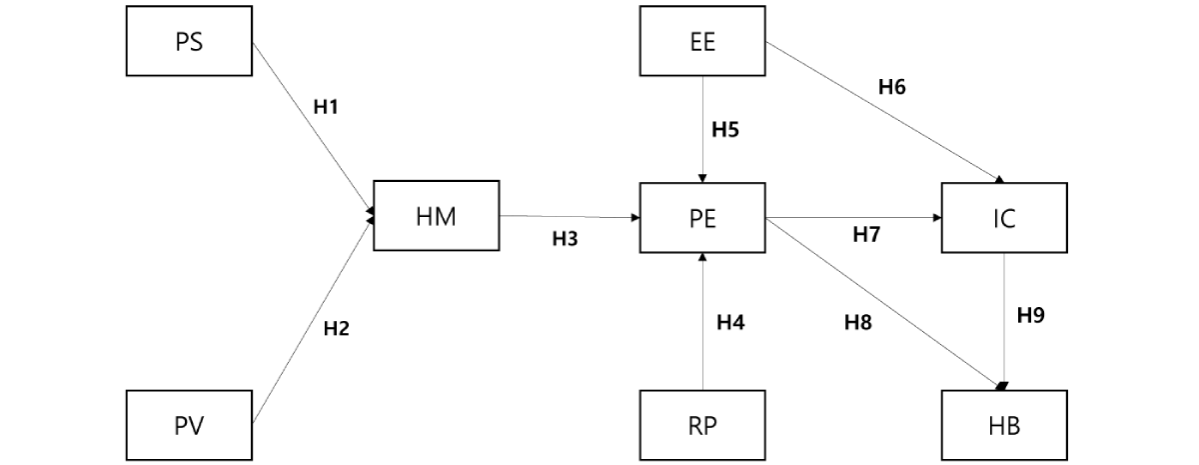
Proposed research model. EE: effort expectancy; HB: habitual use; HM: health motivation; IC: intention of continued use; PE: performance expectancy; PS: perceived severity; PV: perceived vulnerability; RP: risk perception.

We combined UTAUT2 and HBM with the perceived risk regarding personal privacy and habitual use (Figure 1). We adopted certain constructs of HBM to predict individuals’ motivation to engage in healthy behaviors. Perceived vulnerability was defined as the perceived potential for transitioning to CVD if the risk factors for MetS are not controlled. Perceived severity was defined as the socioeconomic burden expected in cases of CVD development. The perceived threat was defined as the sum of perceived vulnerability and perceived severity, and indicated the perceived need to perform healthy behaviors to prevent a severe consequence [30]. We defined health motivation (ie*,* the desire to follow treatment instructions and belief therein) as the degree of motivation for self-health management. We assumed that high perceived severity and perceived vulnerability would lead to strong health motivation.

H1: Individuals with high perceived severity have a strong motivation for health management.

H2: Individuals with high perceived vulnerability have a strong motivation for health management.

We integrated health motivation into the performance expectancy domain of the UTAUT2 model, which indicates the acceptance of wearable devices for health management. According to HBM, individuals with health motivation perceive greater benefits (higher performance expectancy in UTAUT2) than barriers to health behaviors and self-manage their health, which in our case is indicated by the acceptance of wearable health devices [31]. We assumed that individuals with strong health motivation would have high-performance expectancy, that is, they would expect that using wearable devices to manage health is highly productive.

H3: Health motivation positively influences performance expectancy.

Technological advances have led to increased awareness of privacy risks [32] and other risks involved in the adoption and acceptance of wearable devices. Therefore, we added the perception of privacy-related risk to the research model [33]. Risk perception was first proposed by Bauer et al [33] as “a combination of uncertainty and seriousness of outcome involved” [34]. Its effects on performance expectancy are controversial. Some studies have reported that risk perception negatively affects performance expectancy, whereas high personal security during device use is associated with higher performance expectancy [35]. Risk perception may negatively influence performance expectancy, particularly for wearable devices used for health management. Although the perceived risk may be related to several factors associated with the use of information technology [36], we focused on privacy risk, that is*,* the potential loss of control over personal information such as when the personal information of individuals is used without their knowledge or permission [37] because health management using wearable devices requires the collection of personal information. We assumed that a high-risk perception would result in low-performance expectancy.

H4: Risk perception negatively influences performance expectancy.

The main components of UTAUT2 are performance expectancy (ie, an individual’s degree of expectation that they can achieve their goals effectively through the use of technology) and effort expectancy (ie, the degree of expectations regarding the cost or time required to effectively use the technology), which are similar concepts to perceived usefulness and perceived ease of use, respectively, in TAM [17]. However, TAM and UTAUT2 involve conflicting explanations regarding whether effort expectancy directly affects intention to use [38]. According to TAM, familiarity with device use leads to expectations about device use and promotes the continued use of technology (context-agnostic). According to UTAUT2, the intention of continued device use is immediately acquired through familiarity with its use (technology-agnostic) [38]. Based on the latter, we hypothesized that effect expectancy may affect performance expectancy and the intention of continued use. This key concept of the model suggests that the evaluation of the use of wearable health care devices (ie, when performance expectancy exceeds effort expectancy) would lead to a decision on device acceptance [17]. Similar to Son et al [39], we developed items to measure effort expectancy (ie, the degree of perceived ease of use). We hypothesized that effort expectancy would affect performance expectancy and the intention of continued use.

H5: Perceived effort expectancy positively influences performance expectancy

H6: Perceived effort expectancy positively influences the intention of continued use

Previous studies of UTAUT2 have reported high intentions for continued use with high-performance expectancies [23,40,41]. Few individuals reported that wearable health devices did not meet their expectations [24], and 1 study reported that expectations about device performance had a positive relationship with its habitual use [42]. Therefore, we hypothesized that high-performance expectancy may have a positive effect on the intention of continued and habitual use.

H7: Performance expectancy positively influences intention of continued use.

H8: Performance expectancy positively influences habitual use.

In accordance with the evaluation-intention-usage relationship, the intention of continued use positively influences habitual use [43]. We hypothesized that the intention of continued use may lead to habitual use of wearable health devices.

H9: Intention of continued use positively influences habitual use.

### Measurements

The survey tool consisted of 3 parts. The first part evaluated demographic characteristics, including age, sex, occupation, and risk factors for MetS (ie, hypertension, hyperglycemia, hyperlipidemia, and abdominal obesity). The second part contained questions about 3 characteristics of wearable health devices, including the period of use, device type, and main features of the devices. In the final part, items related to each construct were measured.

The measurement items were adopted from previous studies that evaluated health information technology using HBM and UTAUT2: 24 items were derived from HBM and UTAUT2 [44], and 2 items related to risk perception were adopted from Moore and Benbasat [45]. To measure habitual use as a successful behavioral change outcome, we adopted 3 items from UTAUT2 [44]. Because after reviewing 2 qualitative studies evaluating habit formation outcomes [46,47], we found they asked the same questions, such as whether participants felt that their use of devices had become a habit and if the behavioral changes were programmed into their routines. To ensure the validity and reliability of measurement items, we adopted translated items from peer-reviewed Korean studies that used identical models and similar subjects. We translated certain measurement items that were not previously translated. The items were validated by 3 researchers who are fluent in Korean and English. The questionnaire was evaluated in a pilot web-based survey of 17 volunteer graduate students in public health programs, and any ambiguous or confusing questions were modified. Items were scored on a 5-point Likert scale (1=strongly disagree, 2=disagree, 3=neutral, 4=agree, 5=strongly agree).

### Data Collection

A web-based survey used questionnaires consisting of 24 items to evaluate 300 individuals between September 3 and September 7, 2021. We investigated the health motivation and use of wearable devices for health management in middle-aged individuals with risk factors for MetS. For this, we included participants aged ≥40 years with ≥1 risk factor for MetS (hypertension, hyperglycemia, hyperlipidemia, and abdominal obesity) and experience with using wearable devices for health management. The web-based survey was conducted by a web-based panel and field research company. In total, 300 participants (150 females and 150 males) with ≥1 risk factor for MetS were recruited. Table 1 lists the general characteristics of the participants.

**Table 1:** Constructs and questions related to the measurement items.

Construct		Questionnaire
**Perceived severity [48]**		
	PS1^a^	If I develop a cardio-cerebrovascular disease, there will be a social impact.
	PS2	If I develop a cardio-cerebrovascular disease, there will be an economic impact.
	PS3	If I develop a cardio-cerebrovascular disease, there will be an impact on my daily life.
**Perceived vulnerability [49]**		
	PV1^b^	I may develop a cardio-cerebrovascular disease if my health risk factors (hypertension, hyperglycemia, hyperlipidemia, and abdominal obesity) are not managed.
	PV2	My risk of disease may increase if my neighbors have a cardio-cerebrovascular disease.
**Health motivation [50]**		
	HM1^c^	I try to manage my health risk factors (hypertension, hyperglycemia, hyperlipidemia, and abdominal obesity) in my daily life.
	HM2	I try to maintain a healthy lifestyle.
	HM3	The health risk factors (hypertension, hyperglycemia, hyperlipidemia, and abdominal obesity) need to be managed to prevent cardio-cerebrovascular diseases (hypertension, diabetes, and hyperlipidemia).
	HM4	I have an interest in maintaining a healthy lifestyle.
**Effort expectancy [39]**		
	EE1^d^	It is or will be easy to use a wearable device (eg, smartwatch and smart glasses)
	EE2	It is not or will not be very difficult to use a wearable device (eg, smartwatch and smart glasses) proficiently.
	EE3	It is or will be easy to understand how wearable devices (eg, smartwatch and smart glasses) work.
	EE4	It is or will be easy to learn the use of wearable devices (eg, smartwatch and smart glasses).
**Performance expectancy [23]**		
	PE1^e^	The use of wearable devices can improve health status monitoring and management efficiency.
	PE2	The use of wearable devices improves my health.
	PE3	The use of wearable devices helps me achieve my health goals earlier.
**Risk perception (Privacy) [51]**		
	RP1^f^	It would be risky to allow wearable devices to record personal information.
	RP2	Recording personal information on the wearable devices would lead to unexpected problems.
**Intention of continued use [23]**		
	IC1^g^	I plan to continue using wearable devices to record my health status.
	IC2	I will continue to use wearable devices to record my health status.
	IC3	I intend to continue using wearable devices to monitor and manage my health status.
**Habitual use [44]**		
	HB1^h^	It feels natural for me to use wearable devices.
	HB2	I must regularly use wearable devices to monitor my health status.
	HB3	I have developed a habit of using wearable devices to monitor my health status.

^a^PS: perceived severity.

^b^PV: perceived vulnerability.

^c^HM: health motivation.

^d^EE: effort expectancy.

^e^PE: performance expectancy.

^f^RP: risk perception.

^g^IC: intention of continued use.

^h^HB: habitual use.

### Ethics Approval

The study protocol was approved by the Institutional Review Board of Mokpo National University (approval no: MNUIRB-210625-SB-009-01). All participants provided verbal consent for the web-based survey.

### Statistical Analysis

We used structural equation modeling to identify the predictors of habitual use of a wearable health device using the hypothesis model. We evaluated the measurement model and then tested each path of the research model. The reliability and validity of the measurement model were also determined. Descriptive statistics were analyzed using RStudio (version 4.1.2; RStudio Team). Subsequent analyses were performed using SPSS software (version 26; IBM Corp). The reliability of constructs was evaluated using Cronbach α and composite reliability. Cronbach α coefficients of .7-.9 and .5-.7 indicated high and moderate reliability, respectively [52]. A composite reliability of >0.7 indicated good reliability of the construct variables [53]. We examined the validity of construct and latent variables using convergent and discriminant validity. Convergent validity was confirmed if the average variance extracted (AVE) value was ≥0.5 [54]. Discriminant validity was confirmed if the correlations between the 2 constructs were less than the square root of the AVE value [55,56]. The strength of the correlation between each pair of variables was measured using the Pearson correlation test. The structural model fit was used to test the reliability and validity.

Mediation analysis was performed to investigate the direct and indirect effects of performance expectancy on habitual use; the indirect effect was mediated through the intention of continued use. The bootstrapping method was used to determine the significance of the mediating effect, with a 2000-time bootstrapping sample and 95% CI.

## Results

### Demographics and Characteristics Related to Wearable Health Device Use

Table 2 lists the demographics and characteristics related to wearable health device use. Half of the respondents were males, and the other half were females. All were aged 40-69 years, and the proportions of individuals aged 40-49, 50-59, and 60-69 years were similar (34.0%, 35.3%, and 30.7%, respectively). Most respondents worked as machine operators and assemblers (45.7%), followed by managers and professionals (16.7%), and others (16.0%). Hypertension, hyperglycemia, hyperlipidemia, and abdominal obesity were present in 28.1%, 27.9%, 23.5%, and 20.6% of respondents, respectively. In total, 36.7% of respondents had used wearable devices for longer than a year. The most frequently used device was the “Galaxy Watch” (46.9%), followed by the “Mi Band” (24.7%). The most frequently used functions of the devices were monitoring heart rate and blood pressure (34.4%), followed by physical activity (32.3%).

**Table 2:** Demographics and characteristics related to wearable health device use (N=300).

Characteristics			Participants, n (%)
**Demographic characteristics**			
	**Gender**		
		Males	150 (50.0)
		Females	150 (50.0)
	**Age group (years)**		
		40-49	102 (34.0)
		50-59	106 (35.3)
		60-69	92 (30.7)
	**Occupation**		
		Managers and professionals	50 (16.7)
		Machine operators and assemblers	137 (45.7)
		Service workers	26 (8.7)
		Craft and related trades workers	8 (2.7)
		Agriculture, forestry, and fishery workers	3 (1.0)
		Sales workers	28 (9.3)
		Others	48 (16.0)
	**Health status** **(multiple choices may be applicable)**		
		Hypertension	172 (28.1)
		Hyperglycemia	171 (27.9)
		Hyperlipidemia	144 (23.5)
		Abdominal obesity	126 (20.6)
**Characteristics related to wearable health device use**			
	**Period of use**		
		<1 year	190 (63.3)
		≥1 year	110 (36.7)
	**Device type** **(multiple choices may be applicable)**		
		Apple Watch (Apple)	42 (10.9)
		Galaxy Watch (Samsung)	180 (46.9)
		Galaxy Fit (Samsung)	41 (10.7)
		Mi Band (Xiaomi)	95 (24.7)
		Fitbit watch or tracker (Fitbit)	20 (5.2)
		Others	6 (1.6)
	**Main functions** **(multiple choices may be applicable)**		
		Physical activity	226 (32.3)
		Sleep pattern	131 (18.7)
		Heart rate and blood pressure	241 (34.4)
		Stress level	102 (14.6)

### Measurement Model

Table 3 presents the reliability of the measurement model. The item loadings were >0.40 (0.56-0.89) for all items, with a composite reliability of >0.70 (0.85-0.96). The Cronbach α of each factor was >.6 (.61-.84), and the overall Cronbach α was .89. Convergent validity was verified based on the AVE value of >0.5 (0.73-0.89). Table 4 lists the discriminant and construct validity of the measurement model. The discriminant validity was tested using the Fornell-Larcker criterion. The square root of the AVE value (shown in italics) was higher than its highest correlation with other constructs. Pearson correlations with latent variables were measured to evaluate the construct validity. The correlation values were lower than the root of AVE written diagonally on the matrix.

**Table 3:** Construct and composite reliability.

Construct and measured variables		Estimate^a^		SE	Construct reliability	AVE^b^	CR^c^	Cronbach α
		ß^d^	β^e^					
**Perceived severity**						0.823	0.933	.803
	PS1	1	.841	.000	Reference			
	PS2	.846	.699	.076	11.134			
	PS3	.842	.753	.066	12.785			
**Perceived vulnerability**						0.748	0.853	.609
	PV1	1	.782	.000	Reference			
	PV2	.779	.562	.114	6.824			
**Health motivation**						0.771	0.931	.819
	HM1	1	.711	.000	Reference			
	HM2	.912	.670	.068	13.343			
	HM3	.951	.724	.100	9.509			
	HM4	.894	.679	.088	10.137			
**Effort expectancy**						0.824	0.949	.844
	EE1	1	.819	.000	Reference			
	EE2	.917	.701	.076	12.138			
	EE3	.914	.773	.068	13.369			
	EE4	.961	.746	.073	13.201			
**Performance expectancy**						0.726	0.887	.756
	PE1	1	.678	.000	Reference			
	PE2	.990	.678	.092	10.748			
	PE3	.789	.559	.074	10.644			
**Risk perception**						0.781	0.876	.787
	RP1	1	.894	.000	Reference			
	RP2	.870	.726	.061	14.157			
**Intention of continued use**						0.888	0.960	.886
	IC1	1	.865	.000	Reference			
	IC2	.998	.829	.055	18.222			
	IC3	.955	.821	.052	18.222			
**Habitual use**						0.772	0.910	.812
	HB1	1	.805	.000	Reference			
	HB2	.854	.719	.063	13.510			
	HB3	.915	.728	.065	14.054			

^a^*P* value was considered significant at <.001.

^b^AVE: average variance extracted.

^c^CR: composite reliability.

^d^ß : crude path coefficient β

^e^β : standardized path coefficient β

**Table 4:** Discriminant validity^a,b^.

Variable	PS^c^	PV^d^	HM^e^	EE^f^	PE^g^	RP^h^	IC^i^	HB^j^
PS	*0.907*	N/A^k^	N/A	N/A	N/A	N/A	N/A	N/A
PV	0.571	*0.865*	N/A	N/A	N/A	N/A	N/A	N/A
HM	0.473	0.588	*0.878*	N/A	N/A	N/A	N/A	N/A
EE	0.265	0.326	0.423	*0.908*	N/A	N/A	N/A	N/A
PE	0.310	0.520	0.539	0.603	*0.852*	N/A	N/A	N/A
RP	–0.277	–0.125	–0.129	–0.053	0.010	*0.884*	N/A	N/A
IC	0.317	0.423	0.553	0.485	0.755	–0.050	*0.942*	N/A
HB	0.195	0.451	0.582	0.607	0.798	0.068	0.877	*0.879*

^a^Square root of average variance extracted value of each variable and correlation coefficient matrix.

^b^Italicized values represent square root of the average variance extracted value; values below them indicate the correlation coefficients.

^c^PS: perceived severity.

^d^PV: perceived vulnerability.

^e^HM: health motivation.

^f^EE: effort expectancy.

^g^PE: performance expectancy.

^h^RP: risk perception.

^i^IC: intention of continued use.

^j^HB: habitual use.

^k^N/A: not applicable.

### Structural Model

After examining the reliability and validity of the measurement model, the relationships between each construct were tested. The hypotheses were tested by measuring the coefficients of the paths. Table 5 lists the unadjusted and adjusted coefficients and the significance of the results along with the SE. For H1 and H2, the standardized path coefficients of health motivation with perceived severity (H1: β=.243, *P*=.008) and perceived vulnerability (H2: β=.562, *P*<.001) were positive. Health motivation (H3: β=.497, *P*<.001), risk perception (H4: β=.137, *P*=.02), and effort expectancy (H5: β=.558, *P*<.001) had significant positive effects on performance expectancy, while effort expectancy did not have any significant effects (H6: β=−.131, *P*=.16). Intention of continued use was positively related to performance expectancy (H7: β=.848, *P*<.001). Finally, the intention of continued use had a positive effect on habitual use (H9: β=.439, *P*<.001), thereby supporting H9. A mediation analysis was conducted to explore the direct and indirect effects of performance expectancy on habitual use (Table 6). Partial mediation was observed in the path between performance expectancy and habitual use, which was mediated by the intention of continued use. The indirect effect of performance expectancy on habit was significantly mediated by the intention of continued use (=.372, *P*=.03), while performance expectancy had significant direct (=.537, *P*<.001) and total (=.909, *P*<.001) effects on habitual use.

**Figure 2:**
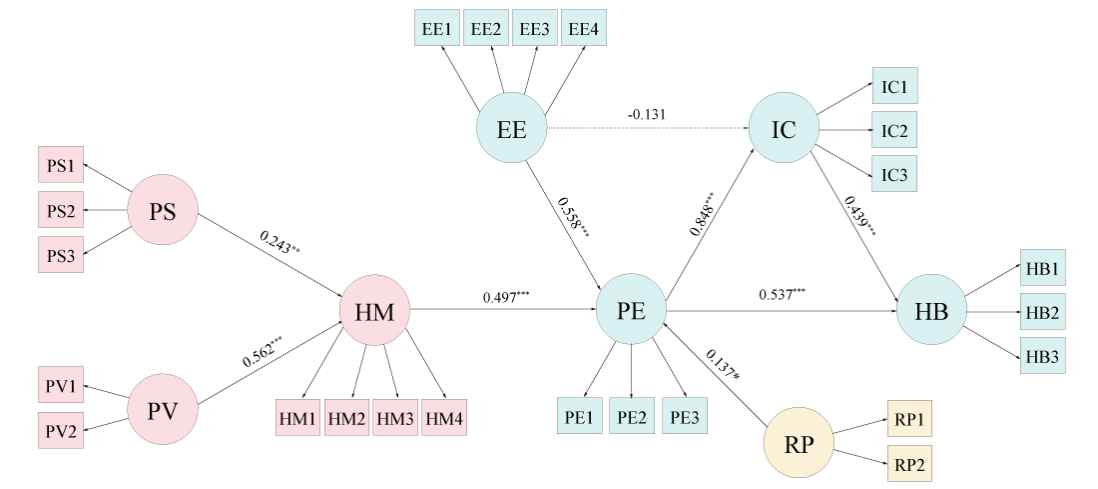
Empirical analysis results of the research model. EE: effort expectancy; HB: habitual use; HM: health motivation; IC: intention of continued use; PE: performance expectancy; PS: perceived severity; PV: perceived vulnerability; RP: risk perception. *P<.01, **P<.005, and ***P<.001.

We present Figure 2 for a more meaningful interpretation of the summary of the research results that conform to the research model. The research results were summarized in Figure 2. Variables from the HBM are colored in red, variables from the UTAUT2 are colored in green, and the variable colored in yellow demonstrates that it is an exogenous variable. The black lines represent the significance of the paths (*P*<.01), and the dotted line represents an insignificant (*P*>.01) path. The fitness indices of the structural model were ideal: chi-square or df (χ²/df)=2.096, goodness-of-fit index=0.871, adjusted goodness-of-fit index=0.841, comparative fit index=0.917, normed fit index=0.854, incremental fix index=0.918, Tucker Lewis index=0.906, and root-mean-square error of approximation=0.061. The model explained 86.6% of the variance in the habitual use of wearable health devices.

**Table 5:** Regression coefficients of pathways in the model.

Path (Hypothesis)	Estimate		SE	Result
	β^a^	*P* value		
H1: Perceived severity → Health motivation	.243	.008	.076	Supported
H2: Perceived vulnerability → Health motivation	.562	<.001	.121	Supported
H3: Health motivation → Performance expectancy	.497	<.001	.070	Supported
H4: Risk perception → Performance expectancy	.137	.02	.032	Supported
H5: Effort expectancy → Performance expectancy	.558	<.001	.060	Supported
H6: Effort expectancy → Intention of continued use	–.131	.164	.101	Not supported
H7: Performance expectancy → Intention of continued use	.848	<.001	.144	Supported
H8: Performance expectancy → Habitual use	.537	<.001	.157	Supported
H9: Intention of continued use → Habitual use	.439	<.001	.112	Supported

^a^Standardized regression coefficient.

**Table 6:** Direct and indirect effects of performance expectancy on habitual use.

Classification IC^a^ (PE^b^ → HB^c^)	Point estimate (β)	Product of coefficients		Bootstrapping (95% CI)			
				Bias-corrected		Percentile	
		SE	*P* value	Lower	Upper	Lower	Upper
Indirect effects	.372	.132	.03	.122	.599	.057	.569
Direct effects	.537	.153	<.001	.284	.861	.301	.898
Total effects	.909	.053	<.001	.787	.998	.791	.999

^a^IC: intention of continued use.

^b^PE: performance expectancy.

^c^HB: habitual use.

## Discussion

### Principal Findings

We evaluated the predictors and the structural relationships thereof related to the habitual use of wearable devices for self-health management in adults aged >40 years and having risk factors for MetS. Our results suggest that high health motivation and high expectations regarding the effectiveness and usefulness of wearable devices for health management (ie, performance expectancy) were associated with habitual use through the voluntary determination of continued use. Performance expectancy, a key component of this process, was influenced by the ease of use and risk perception related to wearable devices, while effort expectancy did not significantly influence the intention of continued use.

Health motivation was influenced by perceived severity and vulnerability, that is, individuals with higher perceived vulnerability and severity had strong health motivation. In this study, users with higher vulnerability were more motivated to manage their health compared to those with greater severity of the disease. Kim et al [57] found that the awareness of the deterioration of their health condition motivated people to take action for self-health management. In a previous study of extended UTAUT2, perceived vulnerability and severity of disease were used as moderators [58]. The authors found that combined perceived usefulness and vulnerability had a greater effect on behavioral intention than combined perceived usefulness and severity of disease; these findings suggest that the recognition of nonspecific risk factors for CVD may more strongly affect the preventive health behaviors than the socioeconomic risks associated with the disease.

Health motivation positively influences performance expectancy. This was the unique path of the research model that connected health motivation from HBM to performance expectancy from UTAUT2. This combined path explains the acceptance of wearable devices for self-health management to lower the risk of perceived vulnerability or disease severity, which is not adequately explained by technology innovation. A similar study from China [23] evaluated the factors that affect behavioral intention to use a smartphone fitness app. In that study, individuals with greater weight loss intentions were more likely to expect that the fitness app would help them lose weight; such individuals were also more likely than others to use the app. A study from South Korea [48] reported that health belief factors, including perceived vulnerability and severity, positively influenced the intention to accept mobile health care services, suggesting that users may seek appropriate tools for self-health management rather than using wearable devices for health care. Therefore, performance expectancy is a belief regarding the health benefits of using wearable devices for self-health management [48].

Although health motivation is essential for building belief in performance expectancy, it is influenced by device-related factors of effort expectancy and risk perception. In this study, effort expectancy, as measured by ease of use, was positively influenced by performance expectancy. Several previous studies have reported that ease of use is closely related to performance expectancy and plays a critical role in adopting new technology [35,38]. However, many recent studies have found that older users are willing to learn when adopting new technologies, such as wearable health devices, and that they are more concerned about the usefulness of the device than its ease of use [28,59,60]. Our results revealed that effort expectancy was not related to the development of the intention to continue using wearable health devices. A previous study reported that older age was associated with more sustained use of technological devices [61]. A qualitative study reported that certain features of wearable health devices, such as alarms and monitoring of daily activities, were effective for developing habitual use of a tracker in older, long-term users [62]. Therefore, device development should focus on its performance rather than its ease of use.

Risk perception (ie, perception of privacy insecurity) was positively associated with performance expectancy, which is inconsistent with some previous results [23,34]. Several studies have explored the paradoxical relationship between the demand for personalized health care through data sharing and the refusal to adopt the technology due to a perceived risk to privacy [63]. The paradox occurs when individuals willingly use health technology despite high privacy concerns [23,63]. For example, Wei et al [23] reported that risk perception was positively related to the behavioral intention to use a fitness mobile app and negatively related to performance expectancy. The authors suggested that some users might have overcome the perceived risks and adopted new technology [23]. Similarly, the privacy calculus theory posits that individuals compare the benefits and risks of behavior [63]. The findings of certain studies are aligned with those of our study as well as the privacy calculus theory. Certain study participants may have perceived the positive health effects of using wearable health devices with data sharing. Furthermore, our results suggest that disclosing information has a positive relationship with the cognitive evaluation of a device’s performance in the post-adoption phase. However, the perception of privacy insecurity may vary depending on the sociocultural context. Therefore, future studies should develop measurement items that are suitable for Korean culture.

The perception of the usefulness and efficiency of health monitoring and management using wearable devices (ie, performance expectancy) had a significant positive effect on the intention of continued and habitual use. Additionally, the intention of continued use was significantly related to habitual use. Our results suggest that the positive recognition of self-health management using wearable devices developed through a conscious evaluation process and may lead to habitual self-health management. Lankton et al [64] explored information technology habits and determinants and found that habitual use predicts continued use of wearable devices. They concluded that the antecedents of habitual behaviors were users’ thoughts on personal relevance and importance of performing activities, satisfaction, prior use, and task complexity in the postadoption stage. Our results suggest that an evaluation of the importance and appropriateness of technology was needed before its automatic use. One study found that several functions of wearable health devices facilitate their habitual use, such as sports activity monitoring, heart rate feedback, step reminders, and activity statistics [65]. The most commonly used functions of wearable health devices by our study participants were monitoring heart rate, blood pressure, and physical activity. The development of devices should focus on advanced features that target the specific health needs of users to manage their risk factors for MetS. This recommendation is supported by our finding that performance expectancy is strongly related to health motivation, the intention of continued use, and habitual use.

### Practice Implications

Our study results have several practical implications. First, perceived vulnerability was strongly related to health motivation and, subsequently, performance expectancy. Therefore, it is necessary to raise awareness about the advantages of the use of wearable devices that can continuously monitor health before CVD development. Second, individuals aged >40 years old with risk factors for MetS in South Korea reported positive perceptions about self-health management using wearable devices. Habitual health management through wearable health devices can be acquired when maintenance strategies are supported by identifying the specific health needs of users. Third, advanced features of wearable health devices should be developed to embed new healthy habits through habitual use of the devices, which can be integrated with self-monitoring. These combined functions may enhance health motivation and performance expectancy, enabling more personalized self-health management.

### Limitations

Several study limitations should be considered. First, we enrolled only middle-aged Koreans with at least 1 risk factor for MetS. This was a conscious decision to reduce the possibility of confounders derived from demographic characteristics by ensuring that the study population was homogenous. Therefore, our results cannot be applied to other groups with different demographic characteristics. Second, more measurement items are needed for individuals with risk factors for MetS, even though they had high reliability and validity in this research. Finally, the proposed integrative model does not include the theoretical backgrounds and characteristics of the original models and instead interprets each construct from a new perspective that is appropriate for the study subject.

### Conclusions

Although many studies have found the effects of wearable devices on health improvement, long-term adaptation and sustained use of wearable health care devices to maintain healthy behaviors remain the challenge for MetS prevention. We conducted an empirical analysis of the predictors of habitual use of wearable health devices by combining HBM, UTAUT2, and perceived risk. Our findings revealed that habitual use of wearable health devices was influenced by users’ expectations that the devices could help effectively manage MetS risk factors. This relationship was partially mediated by the willingness of users to continue the use of the device, and strong motivation was an important predictor of the expectancy of the device’s performance.

The results suggest that developers and health care practitioners should find better ways to meet the performance expectations of the middle-aged population with MetS risk factors. This may involve identifying specific health indicators to be monitored, personal goal setting with detailed behavioral instructions, and reflecting on the context of existing daily routines to form new habits. This also requires implementing easier and more intuitive features that enhance the performance expectancy of wearable health devices, which ultimately influence the long-term and habitual use of the devices.

## Abbreviations

AVE: average variance extracted
CVD: cardiovascular disease
HBM: health belief model
MetS: metabolic syndrome
TAM: technology acceptance model
UTAUT: Unified Theory of Acceptance and Use of Technology
WAT: wearable activity tracker

## Data Availability

The data sets generated and analyzed during the current study are available from the corresponding author upon reasonable request.
